# MORe PREcISE: a multicentre prospective study of patient reported outcome measures in stroke morbidity: a cross sectional study

**DOI:** 10.1186/s12883-022-02634-0

**Published:** 2022-04-20

**Authors:** Amber E. Corrigan, Ben Carter, Alexander Smith, Anna Pennington, Jonathan Hewitt

**Affiliations:** 1 Department of Biostatistics and Health Informatics, Institute of Psychiatry, Psychology & Neuroscience, King’s College London, London, UK; 2 Division of Population Medicine, School of Medicine, Cardiff University, Cardiff, UK; 3 Aneurin Bevan University Health Board, Newport, South Wales UK

**Keywords:** Morbidity, Patient reported outcome, PROM, Stroke

## Abstract

**Background and Purpose:**

The use of patient reported outcomes measures (PROMs) may offer utility that are important for stroke survivors. This study assessed the PROMIS-10, which contains Mental health (MH) and Physical Health (PH) domains, with an additional five stroke specific questions. The aim of this study was to evaluate the association between the MH and PH measures following a stroke and pre-existing health conditions.

**Methods:**

A multicentre prospective cohort study at 19 hospital sites across England and Wales during 2019 was conducted. The association between each PROMIS-10 domain and demographic and health conditions were calculated using a multilevel multivariable linear and present the adjusted mean difference (aMD).

**Results:**

The study enrolled 549 stroke survivors within 14 days of the index event, 232 were women (42.3%) and with a mean age of 72.7 years (SD = 12.9, range 25 to 97). The MH domain was scored as poor in 3.9% of participants, and very good or excellent in almost a half (48.4%). In contrast the PH domain was scored as poor in 39.9%, compared to very good or excellent in 8.5%. The MH domain was associated with pre-existing diabetes (aMD = − 2.01; 95%CI -3.91, − 0.12; *p* = 0.04), previous stroke (aMD = − 3.62; 95%CI -5.86, − 1.39; *p* = 0.001), age (aMD = 0.07; 95%CI: 0.01, 0.14; *p* = 0.037), and female sex (aMD = 1.91; 95%CI 0.28, 3.54; *p* = 0.022). The PH domain was found to be associated with sex (female) (aMD = 2.09; 95%CI 0.54, 3.65; *p* = 0.008) and previous stroke (aMD = − 3.05; 95%CI -5.17, − 0.93; *p* = 0.005).

**Conclusions:**

Almost half of stroke survivors reported poor PH using a PROM with less reporting poor MH. age, and sex were associated with both MH and PH domains, and additionally pre-exising diabetes and stroke were associated with poorer MH. Clinical management offers an opportunity to investigate and intervene to prevent long term poorer health in stroke survivors.

**Supplementary Information:**

The online version contains supplementary material available at 10.1186/s12883-022-02634-0.

## Introduction

The World Health Organisation (WHO) estimates 15 million people suffer from stroke each year, with more than 5 million living with permanent disability [1–3]. Neurological deficits that persist secondary to stroke are heterogeneous and vary across the patient population.

The use of patient reported outcomes measures (PROMs), defined as questionnaires measuring views on health status from the perspective of the patient rather than the clinician, have grown in importance and significance. PROMs offer a way to measure specific functional domains in a way which is meaningful to the patient, encapsulating the patient’s own perspective of their health [4, 5]. To improve the use of PROMs within the stroke community, a consensus Stroke Standard Set of outcome data has been developed which promotes the use of patient reported outcomes as part of a value-based assessment of care [4].

The PROMIS-10 is a patient reported outcome measure which allows components of both physical and mental health to be accessed from the patient perspective [6, 7]. Utilised in stroke, the PROMIS-10, allows consideration of functional and cognitive status post-stroke. It has previously been estimated that around 50% of survivors are chronically disabled [3]. Physically, survivors of stroke have been seen to suffer from impairments in language and speech, swallowing, vision, weakness, and paralysis. Mentally, survivors of stroke have been reported to experience higher levels of anxiety and depression as well as greater levels of cognitive impairment [8–15]. However, the understanding of the impact of symptoms across mental and physical health domains, especially from the patient perspective, is lacking.

### Aims

The primary aim of this study was to assess the quality of life for stroke survivors throughout England and Wales. The primary objective was to determine the prevalence of mental and physical health outcome for stroke survivors, and secondarily, to report if there was any association with clinical and demographic risk factors.

## Methods

### Study design

The study protocol has previously been published [16]. The cohort was recruited between August 2018, and October 2019 from 19 hospital sites with acute and hyper-acute patient facilities in the UK. Data was assessed within 14 days of the index stroke event (the baseline period post-stroke). Data collection was conducted by trained and experienced research staff.

### Ethical approval

All participants provided informed consent to participate for the study. All methods were conducted in accordance with relevant guidelines and regulations. Ethical approval was granted by the NHS Research Ethics Committee – Wales REC 3–18/WA/0299 - Health and Care Research Wales Support and Delivery Centre for all the sites.

### Measures

#### Demographic, lifestyle, and clinical measures

During the baseline assessment the following were assessed: age; sex; stroke type; pre stroke smoking; alcohol consumption; level of care: clinical characteristics which included past medical history (hypertension, diabetes; transient ischemic attacks and prior stroke).

### Patient-Reported Outcome Measures (PROM)

The PROM is a combination of the PROMIS-10 and additional five stroke specific questions. The PROMIS-10 was developed by Cella et. al [7] and has been validated in previous stroke population [4]. The instrument has two domains of physical health (PH) and mental health (MH) and consists of 10 items. Raw domain scores are converted to T-Scores which are normed to the US population. The PROMIS-10 is a well validated and established patient-reported outcome measure [3, 4, 6, 7, 17, 18]. The additional five stroke specific questions were added to include assessment of patient function, which included items on: walking; eating; toileting; dressing; and communication [4, 8, 9, 18]. These additional questions were developed in order to add stroke specific reporting as well as improve functional-capacity reporting within the PROMIS-10. Further, the PROMIS-10 in combination with the five additional questions has been shown to hold value within other common neurological conditions, such as Multiple sclerosis, Parkinson’s disease and Acquired brain injury [18].

### Short-form Montreal Cognitive Assessment (SF-MoCA)

The SF-MoCA is an adapted shorter 10-point version of the 30 item Montreal Cognitive Assessment. This contains three sections, comprising of clock drawing, abstraction and 5-word recall. It is a clinician delivered tool which acts as an indicator of post-stroke cognitive impairment [10].

### Patient Health Questionnaire-9 (PHQ-9)

The PHQ-9 [11] is a widely used self-reported primary care screening tool for depression and has previously been recommended in stroke, the instrument has strong psychometric properties [12].

### Generalised Anxiety Disorder-7 (GAD-7)

Whilst the GAD-7 [13] has not been validated within stroke use, it is a widely used self reported screening tool for generalised anxiety in primary care [14, 15].

### Modified Rankin Scale (mRS)

The mRS [19] is clinician recorded and delineated using the Rankin Focussed Assessment (RFA), a questionnaire that allows global consideration of disability after the occurrence of stroke [20].

### Data analysis

All data analysis were undertaken in Stata version 16.0. The measures were scored using the validated methods. Item missingness (e.g. no more than 30%) within each measure (or domain) were pro-rata mean imputed [21]. Participants with over 30% of missing items were marked as missing.

### Outcomes

The co-primary outcomes were the MH and PH domains of the PROMIS-10. Secondary outcomes included the GAD-7; PHQ-9; mRS, SF-MoCA and the additional 5 stroke specific questions (walking, toileting, dressing, tube feeding and communication).

### Covariates

The following were fitted to assess any association with the outcomes: pre-stroke hypertension, previous TIA, previous stroke, pre-stroke diabetes, male sex, and age.

### Statistical analysis

The association between exposures and outcomes were fitted using a crude and multivariable multilevel linear model, where hospital site was fitted as a random effect. This utilised PH and MH domain T-scores. The multivariable model was adjusted for: age, sex, pre-stroke hypertension, previous stroke event, previous TIA and pre-stroke diabetes diagnosis. Residuals were used to visually inspect the distributional assumptions from each linear model. The analysis presented the mean difference (MD) and adjusted mean difference (aMD) reported with associated 95% CI and *P*-values. We have reported significant differences of 2 (or more) as both statistically and clinically important differences to patients.

## Results

### Study population

From the 19 hospitals 550 participants consented into the study, and one subject withdrew prior to completing the baseline visit assessments, leaving 549. The population consisted of 232 women (42.3%) and 317 men (57.7%) aged between 25 to 97 (mean = 72.7, SD = 12.9) (Supplementary Table 1).

PROMIS-10 MH was poor in 3.9% participants, very good in 29.3% and excellent in 19.1%, and within PH was with poor in 39.9%, very good 7.31% and excellent in 1.19% (Table 1). There were 253 (50.9%) of people were able to walk unaided, 214 (41.4%) who needed help going to the toilet, 220 (42.6%) who needed aid to dress, 31 (6.2%) who needed a tube for feeding and 97 (17.9%) who had problems communicating or understanding (Supplementary Table 2).

**Table 1:** Domain specific breakdown of PRO – Breakdown of Mental and Physical domain of the 10 Question PRO across sub-groups of patients. Sub-groups encompass age groups, sex (male, female), patients who report pre-stroke hypertension, previous TIA, previous stroke and pre-stroke diabetes were reported (*N* = 549). Results are reported as poor, fair, good, very good and excellent. Domain specific cut offs (normed against the US population) as delineated by Hays, Spritzer, Thompson and Cella, 2015; Hays, Schalet, Spritzer and Cella, 2017 – Domain

Table of morbidity outcome domains within PRO Scoring across age, sex and pre-stroke conditions (Hypertension, TIA, stroke and diabetes)												
	Mental Health domain						Physical health domain					
	Poor (< 29)	Fair (30–40)	Good (41–48)	Very Good (49–56)	Excellent (> 56)	Missing	Poor (< 35)	Fair (36–42)	Good (43–50)	Very Good (51–58)	Excellent (< 59)	Missing
**Total**	**20 (13%)**	**101 (18.4%)**	**141 (25.7%)**	**149 (27.1%)**	**97 (17.7%)**	**41 (7.5%)**	**202 (36.8%)**	**122 (22.2%)**	**139 (25.3%)**	**37 (6.7%)**	**6 (1.1%)**	**43 (7.8%)**
**Age**												
18–49	1 (0.2%)	8 (1.4%)	8 (1.4%)	8 (1.4%)	6 (1.1%)	3 (0.5%)	12 (2.2%)	6 (1.1%)	10 (1.8%)	3 (0.5%)	–	3 (0.5%)
50–64	8 (1.4%)	18 (3.2%)	29 (5.3%)	21 (3.8%)	16 (2.9%)	3 (0.5%)	43 (7.8%)	24 (4.4%)	19 (3.4%)	6 (1.1%)	2 (0.4%)	1 (0.2%)
65–74	4 (0.7%)	27 (4.9%)	40 (7.3%)	38 (6.9%)	28 (5.1%(	4 (0.7%)	50 (9.1%)	36 (6.5%)	40 (7.3%)	8 (1.4%)	2 (0.4%)	5 (0.9%)
75–84	5 (0.9%)	37 (6.7%)	41 (7.5%)	53 (9.7%)	33 (6%)	17 (3.1%)	65 (11.8%)	39 (7.1%)	49 (8.9%)	13 (2.3%)	2 (0.4%)	18 (3.2%)
> 85	2 (0.4%)	11 (2%)	21 (3.8%)	27 (4.9%)	14 (2.6%)	14 (2.6%)	31 (5.6%)	15 (2.7%)	20 (26.6%)	7 (9.4%)	–	16 (2.9%)
**Sex**												
Female	10 (1.8%)	45 (8.2%)	60 (10.9%)	59 (10.7%)	34 (6.2%)	24 (4.4%)	88 (16%)	53 (9.7%)	53 (9.7%)	14 (2.6%)	1 (0.2%)	23 (4.2%)
Male	10 (1.8%)	56 (10.2%)	81 (14.7%)	90 (16.4%)	63 (11.4%)	17 (3.1%)	114 (20.7%)	69 (12.6)	86 (15.7%)	23 (4.2%)	5 (0.9%)	20 (3.6%)
**Pre-Stroke Hypertension**												
Yes	9 (1.6%)	58 (10.6%)	73 (13.3%)	78 (14.2%)	52 (9.5%)	19 (3.5%)	100 (18.2%)	76 (13.8%)	71 (12.9%)	19 (3.5%)	1 (0.2%)	22 (4%)
No	11 (2%)	43 (7.8%)	68 (12.4%)	71 (12.9%)	45 (8.2%)	22 (4%)	102 (18.5%)	46 (8.4%)	68 (12.4%)	18 (3.3%)	5 (0.9%)	19 (2%)
**Previous TIA**												
Yes	3 (0.5%)	20 (3.6%)	29 (5.3%)	19 (3.5%)	12 (2.2%)	12 (2.2%)	35 (6.4%)	21 (3.8%)	24 (4.4%)	3 (0.5%)	–	12 (2.2%)
No	17 (3.1%)	81 (14.8%)	112 (20.4%)	130 (23.7%)	85 (15.5%)	29 (5.3%)	167 (30.4%)	101 (18.4%)	115 (20.9%)	34 (6.2%)	6 (1.1%)	31 (5.6%)
**Previous stroke**												
Yes	8 (1.4%)	16 (2.9%	22 (4%)	23 (4.2)	8 (1.4%)	5 (0.9%)	37 (6.7%)	25 (4.6%)	12 (2.2%)	1 (0.2%)	1 (0.2%)	6 (1.1%)
No	12 (2.2%)	85 (15.5%)	119 (21.7%)	126 (22.9%)	89 (16.2%)	36 (6.6%)	165 (30.1%)	97 (17.7%)	127 (23.1%)	36 (6.6%)	5 (0.9%)	37 (6.7%)
**Pre-Stroke Diabetes**												
Yes	6 (1.1%)	30 (5.5%)	38 (6.9%)	36 (6.6%)	13 (2.3%)	1 0.2%)	54 (9.8%)	32 (5.8%)	26 (4.7%)	5 (0.9%)	3 (0.5%)	1 (0.2%)
No	14 (2.6%)	71 (12.9%)	103 (18.8%)	113 (20.6%)	84 (15.3%)	40 (7.3%)	148 (26.9%)	90 (16.4%)	113 (20.5%)	32 (5.8%)	3 (0.5%)	42 (7.7%)

### Demographic and clinical characteristics associated with PROMIS-10

For each analysis the residuals were approximately normally distributed with zero mean and constant variance.

Multivariable analysis for MH was associated with pre-existing diabetes (aMD = − 2.01; 95%CI -3.91, − 0.12; *p* = 0.036, Table 2); female sex (aMD = 1.91; 95%CI 0.28, 3.54; *p* = 0.022); history of stroke (aMD = − 3.62; 95%CI -5.86, − 1.39; *p* = 0.001); and age (aMD = 0.07; 95%CI: 0.01, 0.14; *p* = 0.037). PH were associated with sex (aMD = 2.09; 95%CI 0.54, 3.65; *p* = 0.008), and history of stroke (aMD = − 3.05; 95%CI -5.17, − 0.93; *p* = 0.005). Thus, worse mental health scores were associated previous stroke and pre-stroke diabetes and better mental health scores with male sex and age. Worse physical health scores were associated with previous stroke and better physical health scores were associated with male sex. Previous stroke and pre-stroke diabetes were both statistically significant and clinically important differences.

**Table 2:** Mean difference of hypertension, TIA, previous stroke, diabetes, sex and age associated with PRO mental health domain. The mean difference (MD) and adjusted MD are reported with associated *p*-values and intervals. Statistically significant *p* -values are reported in bold. As a negative score is associated with worse outcome – a negative value indicates a factor resulting in worse outcome

PROMIS-10 Mental Health domain Mean differences						
	MD	***P***-value	(95% CI)	Adjusted MD	***P***-value	(95% CI)
**Pre-Stroke Hypertension**	0.24	0.768	(−1.36, 1.84)	0.65	0.435	(−0.97, 2.27)
**Previous TIA**	−2.01	0.067	(−4.16, 0.14)	−1.67	0.130	(−3.84, 0.49)
**Previous stroke**	−3.75	**0.001**	(−5.95, − 1.56)	− 3.62	**0.001**	(−5.86, − 1.39)
**Pre-Stroke Diabetes**	−2.20	**0.022**	(−4.09, − 0.31)	− 2.01	**0.036**	(− 3.91, − 0.12)
**Sex (Male)**	1.64	**0.048**	(0.01, 3.25)	1.91	**0.022**	(0.28, 3.54)
**Age**	0.06	0.060	(−0.01, 0.12)	0.07	**0.037**	(0.01, 0.14)

The other comparisons were not significant (*p* > 0.05).

### Demographic and clinical characteristics associated with clinical outcomes

For each analysis the residuals were approximately normally distributed with zero mean and constant variance.

Generalised anxiety using GAD-7 was not found to be associated with any demographic or clinical characteristics (*p* > 0.05) (Supplementary Table 3). Worse PHQ-9 scores were associated with pre-stroke diabetes (aMD = 2.34; 95%CI 1.17, 3.50; *p* < 0.001). Lower, and thus better scores, in the PHQ-9 were associated with sex (aMD = − 1.27; 95%CI -2.28, − 0.27; *p* = 0.013), and age (aMD = − 0.08; 95%CI -0.12, − 0.04), p < 0.001). Patients with diabetes had increased depression, male exhibited lower depression, and increasing age had lower depression (Supplementary Table 4).

Better mRS scores were associated with sex (aMD = − 0.26 95%CI -0.48, − 0.04; *p* = 0.018) and worse mRS scores were associated with age (aMD = 0.01; 95%CI 0.01, 0.02; *p* = 0.003) (Supplementary Table 5). Worse SF-MoCA scores were found to be associated with pre-stroke diabetes (aMD = − 0.59; 95%CI -1.15, − 0.03; *p* = 0.039) and age (aMD = − 0.03; 95%CI -0.06, − 0.02; *p* < 0.001) (Supplementary Table 6).

## Discussion

We included 549 stroke survivors and found almost half reported poor physical health. Poorer mental health (MH) outcomes were associated with age, female sex, previous stroke and pre-stroke diabetes and worse physical health (PH) outcomes with female sex and age. The stroke specific questions [4] suggested that stroke survivors had a high degree of stroke specific comorbidity.

The PROMIS-10 has been shown as a feasible instrument in stroke survivors [22–27] and exhibited the components of patient reported morbidity. Particularly when considering the high post-stroke prevalence of PROMIS-10 poor outcomes immediately after stroke. The PROMIS-10 represents an outcome measure that is real and accessible to the stroke survivor. Work by Phillipp et al. also demonstrates the instrument across stroke survivor populations, suggesting it was a valid and reliable instrument in German stroke surviors [28]. Previously, measures accessing quality of life, such as the HRQOL, in stroke, has demonstrated that physical domains are adversely impacted by stroke in diverse communities [29, 30, 31]. Further assessment of the PROMIS-10 in broader populations is needed to integrate the tool to wider clinical populations.

Further, the PROMIS-10 aligns with clinical risk factors of stroke. A pre-stroke diagnosis of diabetes is significantly associated with worse MH PROMIS-10 outcome scores following a stroke. This is mirrored within the association with other outcome measures; pre-stroke diabetes and a worse MoCA and PHQ9 scores across the cohort at the baseline period (Supplementary Table 4 and 6). A diagnosis of diabetes is considered a high-risk factor for stroke and confers worse outcomes in terms of overall morbidity, functional outcomes and readmission or recurrence [32]. Diabetes, as a comorbidity, has been accepted as being highly related to overall outcome [27, 33] and the reflection within the PROMIS-10 measures is not surprising.

The study demonstrated that sex was associated with different outcomes. When using the PROMIS-10 as an outcome measure, male sex was associated with better cognitive and physical domain scores post-stroke (Tables 2 and 3). This was comparable with the modified Rankin scale, (Supplementary Table 3), where male sex was shown to be associated with better physical functioning. This is consistent with the literature; worse post-stroke outcomes have been previously reported across female patients [34–36]. One large scale study of post stroke outcomes including > 19,000 people reported that 3–6 months after stroke women are more likely to experience disability and worse quality of life [37].

**Table 3:** Association of hypertension, TIA, previous stroke, diabetes, sex and age on PRO physical health domain. Both crude and adjusted results are reported with associated *p*-values and intervals. Statistically significant *p*-values are reported in bold. As a negative score is associated with worse outcome – a negative value indicates a factor resulting in worse outcome

PROMIS 10 Physical health domain Mean differences						
	MD	***P***-value	(95% CI)	Adjusted MD	***P***-value	(95% CI)
**Pre-Stroke Hypertension**	−0.22	0.774	(−1.72, 1.28)	0.19	0.808	(− 1.34, 1.72)
**Previous TIA**	−1.65	0.111	(−3.67, 0.38)	− 1.25	0.234	(− 3.31, 0.81)
**Previous stroke**	−3.17	**0.003**	(−5.27,-1.08)	−3.05	**0.005**	**(−** 5.17, −0.93)
**Pre-Stroke Diabetes**	−1.64	0.071	(−3.44, 0.134)	−1.48	0.107	(−3.27, 0.32)
**Sex (Male)**	2.02	**0.009**	(0.50, 3.54)	2.09	**0.008**	**(**0.54, 3.65)
**Age**	0.01	0.724	(−0.05, 0.07)	0.02	0.472	(−0.04, 0.09)

This study offers a novel insight to the use of a PROMIS-10 to assess post-stroke quality of life. We demonstrate the patient outcomes of MH and PH and highlight association with clinical and demographic risk factors. While it must be noted that the PROMIS-10 is not a direct measure of morbidity, adverse scores in this instrument globally is likely to correlate strongly with high morbidity, thus this adds value for the use of PROMs in clinical practice for the feasible measurement of patient-reported outcomes. This is important to consider in the clinical context and may allow a way to understand physical and mental outcomes, that are significant to a patient, in a global manner. By using the PROMIS-10 clinicians may potentially enact a more sensitive measure to indicate morbidity, especially as perceived by the stroke survivor. In turn, this is likely to add understanding of individual patient needs and improve quality of care in conjunction with improvement in quality of life.

This was a large UK wide prospective multicentre study that assessed patient reported outcomes. The limitations to our study are that PROMIS-10 may only be useful in patients with mild to moderate impairment [28, 38]. While 146 of our subjects (26.9%) reported post-stroke aphasia, the degree and severity of impairment was not noted. Therfore, it is unclear if the functional impairment in communication may hinder the completion of the PROMIS-10. The use of patient reported tools in patients that struggle with communication is a current limitation of all self-reporting measures and should continue to be considered. Further, validation and standardisation of the 15-question measure, the PROMIS-10 and five additional reported questions, would be beneficial and allow a more specific measure of physical health, metal health and functional-capacity that is specific to a stroke population.

## Conclusions

PROMs such as the PROMIS-10 offer a feasible way to measure patient reported quality of life.

Future research is needed to compare a wider set of PROMs. Future clinical practice should investigate comorbidity of mental health in stroke survivors.

## Supplementary Information

**Additional file 1: Supplementary Table 1**. Reported Means, Standard deviation and missingness of measures. *Patients were screened for PHQ-9 and GAD-7 with the PHQ-4 as per the methods. The total missingness of the PHQ-9 and GAD-7 are reported in regard to the population as a total, yet not all participants met the requirement for PHQ-9 or GAD-7.

**Additional file 2: Supplementary Table 2**. Proportion of responses to additional five questions, as per Martins et al, in the PRO.

**Additional file 3: Supplementary Table 3**. Association of hypertension, TIA, previous stroke, diabetes, sex and age on clinical outcome measure – GAD7 . Both crude and adjusted results are reported with associated *p*-values and intervals. Statistically significant *p* -values are reported in bold. As a higher score is associated with worse outcome – a positive value indicates a factor resulting in worse outcome.

**Additional file 4: Supplementary Table 4**. Association of hypertension, TIA, previous stroke, diabetes, sex and age on clinical outcome measure – PHQ9 . Both crude and adjusted results are reported with associated *p*-values and intervals. Statistically significant *p* -values are reported in bold. As a higher score is associated with worse outcome – a positive value indicates a factor resulting in worse outcome.

**Additional file 5: Supplementary Table 5**. Association of hypertension, TIA, previous stroke, diabetes, sex and age on clinical outcome measure – mRS Both crude and adjusted results are reported with associated *p*-values and intervals. Statistically significant *p* -values are reported in bold. As a higher score is associated with worse outcome – a positive value indicates a factor resulting in worse outcome.

**Additional file 6: Supplementary Table 6**. Association of hypertension, TIA, previous stroke, diabetes, sex and age on clinical outcome measure – SF-MoCA . Both crude and adjusted results are reported with associated *p*-values and intervals. Statistically significant *p* -values are reported in bold. As a lower score is associated with worse outcome – a negative value indicates a factor resulting in worse outcome.

## Data Availability

The datasets used and/or analysed during the current study are available from the corresponding author on reasonable request.
