# Supplementary material for: MORe PREcISE: a multicentre prospective study of patient reported outcome measures in stroke morbidity: a cross sectional study
Source: BMC Neurol. 2022 Apr 20;22:145. doi: 10.1186/s12883-022-02634-0 (PMC9020003; doi:10.1186/s12883-022-02634-0)
Supplement: Supplementary file 1 — Additional file 1: Supplementary Table 1. Reported Means, Standard deviation and missingness of measures. *Patients were screened for PHQ-9 and GAD-7 with the PHQ-4 as per the methods. The total missingness of the PHQ-9 and GAD-7 are reported in regard to the population as a total, yet not all participants met the requirement for PHQ-9 or GAD-7. [file 12883_2022_2634_MOESM1_ESM.docx]

| **Reported Means, Standard deviation and Missingness of measures** | | | | |
| --- | --- | --- | --- | --- |
|  |  | *Mean (standard deviation)* | *95% Confidence Interval* | *Total Missing* |
| **PROMIS-10 – Physical health** | N=506 | 37.9 (8.7) | (37.1 -38.7) | 43 (7.8%) |
| **PROMIS-10 - Mental health** | N=508 | 46.7 (9.5) | (45.8 -47.5) | 41 (7.4%) |
| **GAD-7** | N=278 | 9.7 (7.5) | (5.9-6.3) | 271 (49.3%)* |
| **PHQ9** | N=261 | 13.9 (9.8) | (12.7-15.1) | 288 (52.4%)* |
| **MoCA** | N=511 | 6.1 (2.8) | (8.8 - 10.6) | 38 (6.9%) |
| **mRs** | N=547 | 2.9 (1.4) | (2.8 - 3.1) | 2 (0.3%) |
