# Supplementary material for: MORe PREcISE: a multicentre prospective study of patient reported outcome measures in stroke morbidity: a cross sectional study
Source: BMC Neurol. 2022 Apr 20;22:145. doi: 10.1186/s12883-022-02634-0 (PMC9020003; doi:10.1186/s12883-022-02634-0)
Supplement: Supplementary file 2 — Additional file 2: Supplementary Table 2. Proportion of responses to additional five questions, as per Martins et al, in the PRO. [file 12883_2022_2634_MOESM2_ESM.docx]

| **PRO – Additional five stroke-specific questions**  **N=549** | |
| --- | --- |
| **Are you able to walk?** |  |
| Able to walk without aid | 263 (47.9%) |
| Able to walk with aid | 156 (28.4%) |
| Unable to walk | 97 (17.6%) |
| Missing data | 33 (6%) |
| **Do you need help from anybody to go to the toilet?** |  |
| Needs aid to toilet | 214 (38.9%) |
| Does not need aid | 302 (55%) |
| Missing data | 33(6%) |
| **Do you need help with dressing/undressing?** |  |
| Needs aid to dress | 220 (40.1%) |
| Does not need aid | 296 (53.9%) |
| Missing | 33 (6%) |
| **Do you need a tube for feeding?** |  |
| Yes | 31 (5.6%) |
| No | 469 (85.4%) |
| Missing | 49 (8.9%) |
| **Do you have problems with communication or understanding?** |  |
| Yes | 92 (16.7%) |
| No | 421 (76.7%) |
| Missing | 36 (6.5%) |
