# Supplementary material for: MORe PREcISE: a multicentre prospective study of patient reported outcome measures in stroke morbidity: a cross sectional study
Source: BMC Neurol. 2022 Apr 20;22:145. doi: 10.1186/s12883-022-02634-0 (PMC9020003; doi:10.1186/s12883-022-02634-0)
Supplement: Supplementary file 3 — Additional file 3: Supplementary Table 3. Association of hypertension, TIA, previous stroke, diabetes, sex and age on clinical outcome measure – GAD7 . Both crude and adjusted results are reported with associated p-values and intervals. Statistically significant p -values are reported in bold. As a higher score is associated with worse outcome – a positive value indicates a factor resulting in worse outcome. [file 12883_2022_2634_MOESM3_ESM.docx]

| **Supplementary Table 3:** | | | | | | |
| --- | --- | --- | --- | --- | --- | --- |
| **Clinical outcome measures - Mean differences – GAD7** | | | | | | |
| **GAD7** | **MD** | **P-value** | **(95% CI)** | **Adjusted MD** | **P-value** | **(95% CI)** |
| **Pre stroke Hypertension** | 0.03 | 0.959 | (-1.12, 1.18) | 0.03 | 0.957 | (-1.16, 1.23) |
| **Pre stroke TIA** | -0.13 | 0.867 | (-1.68, 1.41) | -0.05 | 0.947 | (-1.62, 1.52) |
| **Previous stroke** | -0.31 | 0.702 | (-1.92, 1.29) | -0.28 | 0.734 | (-1.92, 1.35) |
| **Pre stroke Diabetes** | 1.19 | 0.089 | (-0.18, 2.55) | 1.23 | 0.083 | (-0.16, 2.63) |
| **Sex (Male)** | -0.22 | 0.719 | (-1.39, 0.96) | -0.52 | 0.395 | (-1.73, 0.68) |
| **Age** | -0.04 | 0.082 | (-0.09, 0.01) | -0.042 | 0.093 | (-0.09, 0.01) |
