# Supplementary material for: MORe PREcISE: a multicentre prospective study of patient reported outcome measures in stroke morbidity: a cross sectional study
Source: BMC Neurol. 2022 Apr 20;22:145. doi: 10.1186/s12883-022-02634-0 (PMC9020003; doi:10.1186/s12883-022-02634-0)
Supplement: Supplementary file 4 — Additional file 4: Supplementary Table 4. Association of hypertension, TIA, previous stroke, diabetes, sex and age on clinical outcome measure – PHQ9 . Both crude and adjusted results are reported with associated p-values and intervals. Statistically significant p -values are reported in bold. As a higher score is associated with worse outcome – a positive value indicates a factor resulting in worse outcome. [file 12883_2022_2634_MOESM4_ESM.docx]

| **Clinical outcome measures - Mean differences – PHQ9** | | | | | | |
| --- | --- | --- | --- | --- | --- | --- |
| **PHQ9** | **MD** | **P-value** | **(95% CI)** | **Adjusted MD** | **P-value** | **(95% CI)** |
| **Pre stroke Hypertension** | 0.36 | 0.473 | (-0.62, 1.34) | 0.27 | 0.597 | (-0.73, 1.27) |
| **Pre stroke TIA** | 0.25 | 0.710 | (-1.06, 1.56) | 0.22 | 0.746 | (-1.08, 1.52) |
| **Previous stroke** | 0.94 | 0.179 | (-0.43, 2.31) | 0.62 | 0.368 | (-0.74, 1.98) |
| **Pre stroke Diabetes** | **2.38** | **<0.001** | (1.23, 3.53) | 2.34 | **<0.001** | (1.17, 3.50) |
| **Sex (Male)** | -0.82 | 0.111 | (-1.82, 0.19) | -1.27 | **0.013** | (-2.28, -0.27) |
| **Age** | -0.08 | **0.001** | (-0.11, -0.03) | -0.08 | **<0.001** | (-0.12, -0.04) |
