# Supplementary material for: MORe PREcISE: a multicentre prospective study of patient reported outcome measures in stroke morbidity: a cross sectional study
Source: BMC Neurol. 2022 Apr 20;22:145. doi: 10.1186/s12883-022-02634-0 (PMC9020003; doi:10.1186/s12883-022-02634-0)
Supplement: Supplementary file 5 — Additional file 5: Supplementary Table 5. Association of hypertension, TIA, previous stroke, diabetes, sex and age on clinical outcome measure – mRS Both crude and adjusted results are reported with associated p-values and intervals. Statistically significant p -values are reported in bold. As a higher score is associated with worse outcome – a positive value indicates a factor resulting in worse outcome. [file 12883_2022_2634_MOESM5_ESM.docx]

| **Clinical outcome measures - Mean differences – modified Rankin score** | | | | | | |
| --- | --- | --- | --- | --- | --- | --- |
| **mRS** | **MD** | **P-value** | **(95% CI)** | **Adjusted MD** | **P-value** | **(95% CI)** |
| **Pre stroke Hypertension** | 0.13 | 0.238 | (-0.09, 0.34) | 0.07 | 0.559 | (-0.15, 0.29) |
| **Pre stroke TIA** | 0.14 | 0.340 | (-0.14, 0.42) | 0.04 | 0.784 | (-0.25, 0.32) |
| **Previous stroke** | 0.25 | 0.108 | (-0.05, 0.54) | 0.26 | 0.091 | (-0.04, 0.56) |
| **Pre stroke Diabetes** | -0.15 | 0.252 | (-0.41, 0.11) | -0.19 | 0.156 | (-0.44, 0.07) |
| **Sex (Male)** | -0.32 | **0.004** | (-0.53, 0.09) | -0.26 | **0.018** | (-0.48, -0.04) |
| **Age** | 0.02 | **<0.001** | (0.01, 0.02) | 0.01 | **0.003** | (0.01, 0.02) |
