# Supplementary material for: MORe PREcISE: a multicentre prospective study of patient reported outcome measures in stroke morbidity: a cross sectional study
Source: BMC Neurol. 2022 Apr 20;22:145. doi: 10.1186/s12883-022-02634-0 (PMC9020003; doi:10.1186/s12883-022-02634-0)
Supplement: Supplementary file 6 — Additional file 6: Supplementary Table 6. Association of hypertension, TIA, previous stroke, diabetes, sex and age on clinical outcome measure – SF-MoCA . Both crude and adjusted results are reported with associated p-values and intervals. Statistically significant p -values are reported in bold. As a lower score is associated with worse outcome – a negative value indicates a factor resulting in worse outcome. [file 12883_2022_2634_MOESM6_ESM.docx]

| **Clinical outcome measures - Mean differences – SF-MoCA** | | | | | | |
| --- | --- | --- | --- | --- | --- | --- |
| **SF-MoCA** | **MD** | **P-value** | **(95% CI)** | **Adjusted MD** | **P-value** | **(95% CI)** |
| **Pre stroke Hypertension** | -0.62 | **0.009** | (-1.09, -0.15) | -0.27 | 0.276 | (-0.74, 0.21) |
| **Pre stroke TIA** | -0.41 | 0.196 | (-1.05, 0.22) | -0.10 | 0.756 | (-0.73, 0.53) |
| **Previous stroke** | -0.56 | 0.092 | (-1.22, 0.09) | -0.47 | 0.157 | (-1.12, 0.18) |
| **Pre stroke Diabetes** | -0.69 | **0.015** | (-1.25, -0.14) | -0.59 | **0.039** | (-1.15, -0.03) |
| **Sex (Male)** | 0.26 | 0.289 | (-0.22, 0.73) | 0.12 | 0.663 | (-0.37, 0.59) |
| **Age** | -0.04 | **<0.001** | (-0.06, -0.02) | -0.03 | **<0.001** | (-0.06, -0.02) |
